# Safety and efficacy in pediatric secondary intraocular lens implantation, in-the-bag versus sulcus implantation: a multicenter, single-blinded randomized controlled trial

**DOI:** 10.1186/s13063-023-07411-z

**Published:** 2023-06-09

**Authors:** Yingshi Zou, Ling Jin, Bo Qu, Hui Chen, Mingbing Zeng, Xia Li, Xinhua Liu, Lixia Luo, Zhenzhen Liu, Yizhi Liu

**Affiliations:** 1 State Key Laboratory of Ophthalmology, Zhongshan Ophthalmic Center, Guangdong Provincial Key Laboratory of Ophthalmology and Visual Science, Guangdong Provincial Clinical Research Center for Ocular Diseases, Sun Yat-Sen University, 7# Jinsui Road, Guangzhou, 510000 China; 2 Hainan Eye Hospital, Zhongshan Ophthalmic Center, Sun Yat-Sen University Haikou, Hainan, China; 3 The First Affiliated Hospital of Guangxi Medical University, Nanning, Guangxi China; 4 Shenzhen Eye Institute, Shenzhen Eye Hospital, Jinan University, Shenzhen, China

**Keywords:** Pediatric aphakia, Secondary intraocular lens implantation, Glaucomarelated, Adverse events, In-the-bag IOL implantation, Sulcus IOL implantation

## Abstract

**Background:**

Treatment of pediatric cataract remains challenging because of the extremely high incidence of postoperative adverse events (AEs), especially the AEs related to the locations of secondary implanted intraocular lens (IOL). There are two common locations for secondary IOL implantation in pediatric aphakic eyes: ciliary sulcus or in-the-bag implantation. However, there are currently no large, prospective studies comparing complication rates and visual prognosis of in-the-bag versus ciliarysulcus secondary IOL implantation in pediatric patients. Whether or how much secondary in-the-bag IOL implantation benefits the pediatric patients more than sulcus implantation and deserves to be performed routinely by surgeons remains to be elucidated. Here, we describe the protocol of a randomized controlled trial (RCT) designed to evaluate the safety and efficacy of two approaches of IOL implantation in pediatric aphakia.

**Methods:**

The study is a multicenter, single-blinded RCT with 10 years of follow-up. Overall, a minimum of 286 eyes (approximately 228 participants assuming 75% have two study eyes) will be recruited. This study will be carried out in four eye clinics across China. Consecutive eligible patients are randomized to undergo either secondary in-the-bag IOL implantation or secondary sulcus IOL implantation. Participants with two eyes eligible will receive the same treatment. The primary outcomes are IOL decentration and the incidence of glaucoma-related AEs. The secondary outcomes include the incidence of other AEs, IOL tilt, visual acuity, and ocular refractive power. Analysis of the primary and secondary outcomes is to be based on the intention-to-treat and per-protocol analysis. Statistical analyses will include the *χ*^2^ test or Fisher’s exact test for the primary outcome, mixed model and generalized estimated equation (GEE) model for the secondary outcome, Kaplan–Meier survival curves for the cumulative probability of glaucoma-related AEs over time in each group.

**Discussion:**

To the best of our knowledge, this study is the first RCT to evaluate the safety and efficacy of secondary IOL implantation in pediatric aphakia. The results will provide high-quality evidence for the clinical guidelines for the treatment of pediatric aphakia.

**Trial registration:**

ClinicalTrials.gov NCT05136950. Registered on 1 November 2021.

## Introduction

### Background

Treatment of pediatric cataract, one of the leading causes of childhood blindness globally, remains challenging because of the extremely high incidence of postoperative adverse events (AEs) [1–4]. Especially, the AEs related to the locations of secondary implanted IOL, such as iris-related inflammation, glaucoma-related AEs, and IOL positional stability potentially affect patients’ prognosis [5–9].

Theoretically, in-the-bag implantation of IOL prevents contact with the iris and may reduce the risk of AEs [10, 11]. However, due to the difficulties in performing secondary in-the-bag implantation in pediatric aphakic eyes, secondary IOL implantation with ciliary sulcus fixation in these patients is considered acceptable [12–15].

We and other investigators have demonstrated high feasibility of secondary in-the-bag IOL implantation in pediatric aphakic eyes [1, 7, 16–19]. In our prospective comparative study of 355 pediatric eyes undergoing secondary IOL implantation, we investigated the 3-year outcomes of these two methods and found that compared to ciliary sulcus secondary IOL implantation, in-the-bag IOL implantation reduced AEs and yielded better IOL centration and best corrected visual acuity (BCVA) for pediatric aphakia [1]. However, there have been no large, prospective cohort studies adjusting for important determinants of AEs and comparing outcomes of in-the-bag versus ciliary-sulcus secondary IOL implantation.

Whether or how much secondary in-the-bag IOL implantation benefits the pediatric patients more than sulcus implantation and deserves to be performed routinely by surgeons remains to be elucidated.

### Objectives

#### Main objective

The main objective is to compare the safety of in-the-bag versus ciliary-sulcus secondary intraocular lens (IOL) implantation in pediatric aphakia, including IOL decentration and the incidence of glaucoma-related adverse events.

#### Secondary objective

The secondary objective is to compare the efficacy of in-the-bag versus ciliary-sulcus secondary intraocular lens (IOL) implantation in pediatric aphakia, including the incidence of other adverse events, IOL tilt, visual acuity (VA), and ocular refractive power.

## Methods

### Trail design

**Fig. 1:**
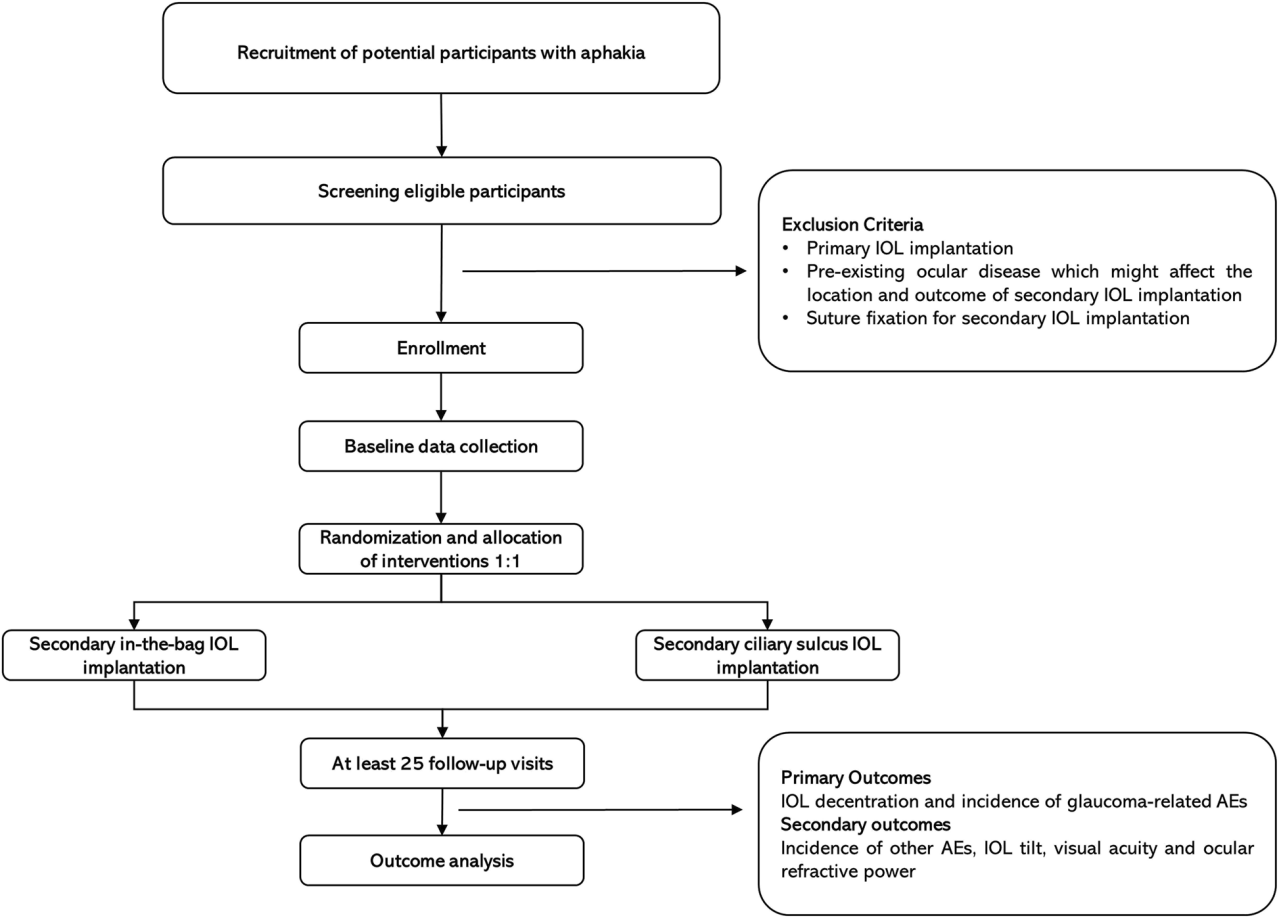
Study flow chart

This protocol follows the Standard Protocol Items for Clinical Trials (SPIRIT 2013) [20]. The study flow chart is shown in detail in Fig. 1.

A Study design: prospective, multicenter, superiority, parallel-group, single-blinded, and randomized controlled trial
B Intervention groups: consecutive eligible patients are randomized to undergo either secondary in-the-bag IOL implantation (capsular group) or secondary sulcus IOL implantation (sulcus group)

Study participants may have one or two study eyes. Study participants with two study eyes eligible will receive the same treatment. Further details on randomization are located in the “Randomization” section.

For both intervention groups, the surgeries are described in the “Interventions” section.

C Sample size
  A minimum of 286 eyes (approximately 228 study participants assuming 75% have two study eyes)
D Duration of follow-up
  Primary outcome: 1 year
  Total follow-up: 10 years
E Follow-up schedule
  Years 1: follow-up visits occur 1 day, 1 week, 1 month, 3 months, 6 months, and 1 year postoperatively
  Years 2 to 10: follow-up visits occur every 6 months

### Study setting

This study will be carried out in four eye clinics across China, including (1) Zhongshan Ophthalmic Center (ZOC), Sun Yat-sen University; (2) Hainan Eye Hospital, Zhongshan Ophthalmic Center, Sun Yat-sen University (HNZOC); (3) the First Affiliated Hospital of Guangxi Medical University; and (4) Shenzhen Eye Hospital.

### Informed consent

On attending the screening clinic, potentially eligible participants and their parents (or legal guardians) will be approached by a trained research coordinator to determine whether they want to participate in the trial. If they express their interest, the researcher will gain informed consent and recruit the patient. Written informed consent will be obtained from the parent or legal guardian before performing the standard clinical surgical treatment. Once the patient is enrolled in the study, he/she will be assigned to either the capsular group or the sulcus group. When the participants reach 18, they should re-consent.

### Study participant eligibility criteria

Patients will need secondary IOL implantation if all the following findings and conditions are met:

- Age over 18 months for unilateral aphakia and over 24 months for bilateral aphakia
- Axial length falls within the mean plus or minus two standard deviations of the age-matched healthy counterparts
- Corneal diameters between 9.5 and 12.5 mm

#### Inclusion criteria

Patients of either gender will be eligible for the study if all the following findings and conditions are met:

- Aged between 18 months and 14 years
- Had a primary diagnosis of congenital cataract
- Underwent unilateral or bilateral cataract extraction between the ages of 2 and 24 months
- Pediatric eyes with sufficient space of anterior segment and volumized Soemmerring’s ring

#### Exclusion criteria

Patients will be excluded from the study if they meet any of the following criteria:

- Primary IOL implantation
- Pre-existing ocular disease which might affect the location and outcome of secondary IOL implantation (including and not restricted to microphthalmia, microcornea, microcornea, corneal opacity, pseudopterygium, iris anomaly, glaucoma diagnosed before cataract extraction, uveitis, persistent fetal vasculature or trauma)
- Suture fixation for secondary IOL implantation

### Interventions

Participants will be randomly divided into two arms in a 1:1 ratio. Surgery in two arms will be performed by qualified surgeons using the standard operating procedure of IOL implantation [1, 16].

After a conjunctival peritomy is made, a 3.2-mm superior scleral tunnel incision will be created. Sodium hyaluronate will be used to maintain the anterior chamber. Then, surgery will proceed in the different groups as per random allocation. All operations will be videotaped in their entirety.

#### Secondary in-the-bag IOL implantation (capsular group)

The Soemmerring’s ring will be reopened intraoperatively to remove proliferated lens material. After the capsular bag is reestablished with sodium hyaluronate, an IOL will be implanted into the peripheral capsular bag.

#### Secondary ciliary sulcus IOL implantation (sulcus group)

The Soemmerring’s ring will be reopened intraoperatively to remove proliferated lens material. Then, an IOL will be implanted into the ciliary sulcus.

The subsequent steps include (secondary) capsulorhexis of the posterior capsule, anterior vitrectomy, irrigation, and aspiration. The tunnel incision is sutured with 10–0 nylon sutures. All patients will be received subconjunctival dexamethasone (2 mg) before the surgery is completed.

#### IOL selection

- IOL styles: the style of IOL used for capsular bag implantation is single-piece hydrophobic spheric monofocal IOL (SA60AT, Alcon Laboratories, America), and the style of IOL used for sulcus implantation is three-piece hydrophobic spheric monofocal IOL (AR40e, Sensar, America).
- IOL power: targeted postoperative refraction range from + 4 diopters to emmetropia will be recommended according to age at secondary IOL implantation and/or the ocular refraction of the contralateral eye.
- IOL power adjustment for sulcus implantation: When the predicted power is between 11.5 and 30D, a corresponding reduction of 0.50–2.50D is made for a ciliary sulcus implanted IOL according to the unpublished refractive data from our cohort.

#### Postoperative eyedrops regimen

The postoperative eyedrops regimen to be followed for each group is the same. Postoperatively, Tobradex eye drops (tobramycin 0.3%, dexamethasone 0.1%, Alcon) will be used 6 times per day, and Tobradex eye ointment (tobramycin 0.3%, dexamethasone 0.1%, Alcon) will be applied once per night for 2 weeks. From 2 weeks to 1 month postoperatively, the eye drops will be used 4 times per day. For the second postoperative month, the patient will be switched to pranoprofen eye drops 4 times per day (Senju Pharmaceutical Co., Ltd., Osaka, JP) [16]. The medication regimen will be adjusted in time according to the individual postoperative situation. For example, for pediatric patients with postoperative glaucoma, topical lowering intra-ocular pressure drops will be added after ruling out the contraindications of local medication.

### Criteria for discontinuing or modifying allocated interventions

The end point of the study is to reach 10 years of follow-up. If intraoperative AEs occur during the study, including hyphema, vitreous hemorrhage, iris or vitreous prolapse, and iatrogenic injury of iris and, participants will receive in-time treatment, and the follow-up will continue. The AEs will be reported to the researchers and the ethics committees as required indicating expectedness, seriousness, severity, and causality.

### Strategies to improve adherence to interventions

We will introduce the purpose of the study and the interventions in detail and actively respond to any questions, and the participants will be closely followed up and deal with adverse events in time if happened.

### Relevant concomitant care permitted or prohibited during the trial

All relevant treatments should be used under the judgment of the clinician. The reason and detailed usage of additional medication should be recorded on the CRF.

### Outcomes

#### Primary outcomes

The primary outcomes include IOL decentration and the incidence of glaucoma-related AEs by 1 year postoperatively.

1. Glaucoma-related AEs are defined using the Infant Aphakia Treatment Study: “comprising ocular hypertension, pupil block, and glaucoma” [19].
  1. Ocular hypertension is defined as intraocular pressure (IOP) ≥ 21 mm Hg.
  2. Transient ocular hypertension is defined as elevated IOP which is resolved with cessation of topical steroids. Cases which do not resolve are classified as persistent ocular hypertension.
  3. Pupil block is defined as ocular hypertension in the context of a history of obstruction of aqueous flow through the pupil.
  4. Glaucoma is defined using the 2001 British Infantile and Childhood Glaucoma (“BIG eye”) study group taxonomy: “the presence of a combination (more than one) of clinical signs consistent with high intraocular pressure (≥ 21 mmHg), such as high pressure, optic disc cupping ≥ 0.3 or disc asymmetry ≥ 0.2, progressive disc cupping, buphthalmos, enlarged corneal diameter, corneal edema, Descemet’s membrane splits/Haab’s striae, visual field defects, or progressive myopia.
2. IOL decentration will be measured using anterior segment optical coherence tomography (AS-OCT, CASIA2, Tomey Corporation, Japan) or Scheimpflug tomography (Pentacam HR 70900, Oculus GMbH, Germany) as previously described [21]. The details can be found in the “Testing procedures” section.

#### Secondary outcomes

The secondary outcomes include the following:

1. Incidence of glaucoma-related AEs by 6 months, 3 years, 5 years, and 10 years postoperatively
2. IOL decentration by 6 months, 3 years, 5 years, and 10 years postoperatively.
3. Incidence of other AEs by 6 months, 1 year, 3 years, 5 years, and 10 years postoperatively. Other AEs include IOL dislocation, iris synechia, corectopia and/or discoria, visual axis opacification, corneal endothelium decompensation, and retinal detachment. It will be recorded separately using criteria previously reported [22, 23].
4. IOL tilt by 6 months, 1 year, 3 years, 5 years, and 10 years postoperatively.
5. VA and ocular refractive power by 6 months, 1 year, 3 years, 5 years, and 10 years postoperatively.

### Participant timeline

The time schedule for enrollment, interventions, assessments, and visits for the participants is summarized in Table 1.

**Table 1:** The schedule of enrollment, interventions, and assessments

Time point	Study period							
	**Enrollment**	**Baseline visit**	**Intervention**	**Follow-up visits**				**Close-out**
	*** − t***_**1**_	***t***_**1**_		***t***_**2**_	***t***_**3**_	***t***_**4**_	**etc**	***t***_**25**_
**Enrollment**								
**Eligibility screen**	X							
**Informed consent**	X							
**History information**	X							
**Baseline testing**	X							
**Allocation**		X						
**Interventions**								
***Secondary in-the-bag IOL implantation***		X						
***Secondary ciliary sulcus IOL implantation***		X						
**Assessments**								
***Vital signs***	X	X	X	X	X	X	etc	X
***Systemic examinations***		X						
***Visual acuity***	X	X		X	X	X	etc	X
***Ocular refractive power***	X	X			X	X	etc	X
***Intraocular pressure***	X	X		X	X	X	etc	X
***Slit lamp***	X	X		X	X	X	etc	X
***Cornea endothelium count***		X			X	X	etc	X
***Ocular biological measurement***		X			X	X	etc	X
***Slit-lamp photography***		X		X	X	X	etc	X
***Pentacam***		X			X	X	etc	X
***CASIA2***		X			X	X	etc	X
***Fundus photography***		X			X	X	etc	X
***Optical coherence tomography***		X			X	X	etc	X

### Sample size

According to the Zhongshan Ophthalmic Center’s outpatient record system, (1) the one-year incidence of glaucoma-related AEs in sulcus implantation is 12%. Assuming the in-the-bag implantation would reduce the absolute incidence to 0.7%, to achieve 90% power with a two-sided alpha of 0.0492 (the interim analysis at 6 months postoperatively spending alpha of 0.0054 by O’Brien-Fleming approach), 114 eyes are needed for each group (total of 228 eyes) using Fisher’s exact test with pooled variance. Allowing for a 20% loss to follow-up, 143 eyes should be needed in each group. Assumptions: % of bilateral = 75%, inter-eye correlation among bilateral patients = 0.5. For bilateral patients, two eyes have the same type of surgery. The number of patients (*X*) per group can be calculated by solving the following equation: *X* × 0.75 × 2/(1 + 0.5) + *X* × 0.25 = 143, *X* = 114 patients per group, a total of 228 patients. The sample size is calculated by PASS 16.0 (NCSS, LLC, USA). (2) IOL decentration in 3-dimension in the capsular group is 0.38 mm while in the sulcus group, it is 0.56 mm. Group sample sizes of 106 and 106 achieve 90% power to detect the mean difference of 0.18 between two trial arms with a common standard deviation for both groups of 0.40 and with a two-sided alpha of 0.0492 using a two-sided two-sample equal-variance *t*-test (same interim analysis as above for the 1-year incidence of glaucoma-related AEs). Allowing for a 20% loss to follow-up, 133 eyes should be needed in each group. Assumptions: % of bilateral = 75%, inter-eye correlation among bilateral patients = 0.5. For bilateral patients, two eyes have the same type of surgery. The number of patients (*X*) per group can be calculated by solving the following equation: *X* × 0.75 × 2/(1 + 0.5) + *X* × 0.25 = 133, *X* = 106 patients per group, a total of 212 patients. The sample size is calculated by PASS 16.0.

Considering the sample size calculated by the two primary outcomes, the largest one is selected: 114 patients per group, a total of 228 patients.

### Recruitment

Consecutive potentially eligible participants will be identified in four eye clinics including ZOC, HNZOC, the First Affiliated Hospital of Guangxi Medical University, and Shenzhen Eye Hospital. Participants meeting the inclusion criteria will be enrolled in this study.

### Randomization

Stratified block randomization will be applied for this trial. The eligible participants stratified by site who consented will then be divided into two strata according to secondary intraocular lens implantation in the unilateral eyes or bilateral eyes. Participants in each stratum will be randomized (1:1) to receive the assigned treatment from either secondary in-the-bag IOL implantation or secondary sulcus IOL implantation. An independent statistician without involvement in the study will generate the randomization sequence for each stratum using an online randomization number generator (http://www.randomization.com).

1. Study participants with one study eye will be randomly assigned to one of the treatment groups.
2. Study participants with two study eyes (both eyes are eligible):
  If capsular surgery is applicable for both eyes, study participants will be randomly assigned to one of the treatment groups, and both eyes will receive the same treatment.
  If capsular surgery is applicable for only one eye, which will be randomly assigned to one of the treatment groups, the contralateral eye will receive sulcus surgery or other appropriate treatment.

### Masking

This is a single-blinded study, and participants are masked to the group assignment. Allocation codes are concealed before the secondary IOL implantation. Participating surgeons will be informed of the study group allocation just prior to the implantation of the IOL. The trial statistician who is responsible for the statistical analysis of the study is not involved in the assignment of treatments to participants. Research assistants determining participant eligibility, examiners, and outcome assessors cannot be masked to the group allocation because treatment can be identified from medical image.

### Data collection

Twenty-five visits including baseline visits over 10 years are planned, with follow-up examinations scheduled at 1 day, 1 week, 1 month, 3 months, 6 months, 12 months for the first year, and every 6 months thereafter.

Every time close to the scheduled visit, our research assistants will sincerely remind participants by text message or phone call. Besides, we offer a partial fee waiver for participant inspections.

#### History information

The following patient information will be collected: sex, age at secondary IOL implantation, age at the cataract extraction, laterality of IOL implantation, operation interval, systemic and ocular history, ocular and systemic comorbidities, and eye symptoms.

#### Baseline testing

All participants will be required to undergo systemic and ocular preoperative examinations. The assessment will be carried out by personnel who are masked to the study group assignment.

- Vital signs
- Systemic examinations: blood routine, biochemical tests, chest radiography, and electrocardiogram
- Ocular examinations: visual acuity, ocular refractive power, intraocular pressure (IOP), slit lamp, cornea endothelium count, ocular biological measurement, slit-lamp photography, Pentacam, fundus photography, and optical coherence tomography

#### Testing procedures

##### Assessment of visual acuity

Visual acuity will be evaluated with an Early Treatment Diabetic Retinopathy Study (ETDRS) chart. For those who are unable to use the ETDRS chart, visual acuity is evaluated with Lea Symbol Chart or Teller Acuity Cards.

All decimal visual acuity outcomes will be converted to logarithm of the minimum angle of resolution (logMAR) units for the statistical analyses.

##### Assessment of ocular refractive power

Ocular refractive power will be measured by an auto refractometer (KR800, TOPCON, Japan) after cycloplegia with 0.5% Tropicamide.

The average sphere, cylinder, and axis will be recorded.

##### Assessment of IOP

IOP is measured by a non-contact tonometry (NT-510, Nidek, Japan). If the participants cannot be coordinated with NCT, an iCare HOME tonometer (TA022, Icare Oy, Finland) is available. If the level of IOP is higher than normal, a Goldmann applanation tonometry (AT900, Haag Streit, Switzerland) will be used for further confirmation.

##### Ocular biological measurement

Includes central corneal thickness, anterior chamber depth, and axial length, measured by IOL-Master (IOLmaster 700, Carl Zeiss Meditec, Germany).

##### Slit-lamp photography

A slit-lamp photography system (BQ900, Haag Streit, Switzerland) will be used to capture retroillumination photos after dilation.

Standardized settings: magnification of × 10, beam height 4 mm, beam width 1 mm, flash off, illumination intensity 80%, and aperture 2.

##### Measurements of IOL position

IOL position (tilt and decentration) will be measured using CASIA2 or Pentacam connected to a digital Scheimpflug rotational camera as previously described [21]. After pupil dilation, two images at slit angles of 90° and 180° will be taken, and 3-dimensional imaging of the IOL position will also be measured.

The anterior and posterior surfaces of the cornea and both IOLs will be marked in the digital image to determine the visual axis of the eye and the optical axis of the IOL.

The tilt of the optical axis of the IOL relative to the visual axis and the distance between the IOL vertex and the visual axis will be calculated using Image-Pro plus 6.0 (Media Cybernetics, MD, USA).

##### Cornea endothelium count

A noncontact specular microscope (NSP-9900, Konan Medical Inc., Japan) will be used to measure corneal endothelial cell density.

##### Fundus photography

Images centered on the disc and macula will be taken for each eye using fundus cameras (Nonmyd WX3D, KOWA, Japan; TRCNW400, TOPCON, Japan) after dilation.

Optic disc progression will be assessed by comparing the stereo photographs taken at baseline and the stereo photographs obtained at the follow-up examination and is defined by any of the following: (1) enlargement of vertical cup-to-disc ratio (VCDR), (2) neuroretinal rim notching (incidence or enlargement), (3) wedge-shaped RNFL defects (incidence or enlargement), or (4) disc hemorrhage, if not related to myopic changes.

##### Optical coherence tomography

Both a swept-source OCT (DRI-OCT Triton, TOPCON, Japan) and a spectral domain OCT (Cirrus 5000 HDOCT, Carl Zeiss Meditec, USA) devices will be used after dilation.

Image quality should be higher than 60 and 5 in DRI-OCT triton and Cirrus 5000 HD-OCT, respectively.

### Data management

#### Case report forms (CRFs)

The clinical coordinator will use paper CRFs for data collection. Eligible patients enrolled in the study will have individual patient CRF binders. The baseline CRFs should be completed on the first visit, signed by the investigator, and stored in the patient’s CRF binder. Investigators are required to complete CRFs at each visit for all enrolled participants after the completion of the research.

#### Data input and verification

The form data will be recorded and keyed into the EDC system and managed by data managers after data monitoring. The EDC system is secured digitally by investigators, and the study team will have access to the research data. If the data managers have questions about the data, investigators are required to respond to these questions on time, and the data managers will be allowed to verify and modify the ultimate data.

#### Data locking

The confirmed dataset will be locked by the principal investigator, monitor, and statistician for statistical analysis. The verified data cannot be modified unless problems arise after data locking, and investigators could modify data appropriately and record it after confirmation.

### Statistical analysis

#### Statistical methods

The normality of continuous data is to be checked using the Shapiro–Wilk test and histogram. Continuous data are to be summarized as the mean ± standard deviation, median, or interquantile range (IQR). Analysis of the primary and secondary outcomes is to be based on the intention-to-treat (ITT) and per-protocol (PP) analyses, and missing data will be imputed by multiple imputation or other appropriate methods. In the ITT analysis, participants are evaluated on the basis of the group to which they were randomly assigned, regardless of whether they actually received the surgery. In the PP analysis, participants are evaluated on the basis of the group to which they actually received the surgery to truly reflect the difference between the two groups. Paired *t* test will be used to compare pre- vs. postoperative visual acuity (in logMAR), spherical equivalent aberration, IOL tilt, and decentration at each time point after surgery. Comparison between the two treatment groups will be performed using a mixed model for continuous outcomes and a generalized estimated equation (GEE) model for categorical outcomes. The incidence of glaucoma-related AEs (primary outcome) by 1 year will be compared using the *χ*^2^ test or Fisher’s exact test. Kaplan–Meier survival curves are to be constructed to show the cumulative probability of glaucoma-related AEs over time in each group, and the log-rank test is used to compare the time to incidence of glaucoma-related AEs between the two study groups. Unadjusted and adjusted hazard ratio (HR) of glaucoma-related AEs and 95% CIs will be estimated by univariable and multivariable Cox proportional hazards model, respectively. Study group, stratification variable (laterality), and other baseline variables with *P* < 0.20 in the univariable analysis will be included in the multivariable model. The inter-eye correlation will be adjusted for all analyses including both eyes from the same subject.

A two-sided *p* < 0.0054 for interim analysis and *p* < 0.0492 for the primary analysis is considered to be statistically significant. All statistical analysis will be performed using Stata 16 (StataCorp, College Station, TX, USA) and other appropriate software.

#### Interim analyses

We plan to perform one interim analysis at 6 months postoperatively in the intention-to-treat population, up to half of the objective samples.

### Data monitoring

The data monitoring committee (DMC) members include Prof. Ying Han, Prof. Guishuang Ying, and Prof. Nathan Congdon, who have no competing interests. The responsibility of the DMC is to review the study design and study documents before the study starts of the study to identify any problems that might affect future data analysis or participants’ safety.

### Research-related risks and risk management

There is a potential risk of complications associated with IOL implantation surgery.

Intraoperative complications will be managed accordingly by the surgeon, and postoperative complications will be managed by the ophthalmologist who carries out the ocular assessment.

### Auditing trial conduct

The principle investigator, the study team, the independent DMC, and the ethics committee will meet once a year through the trial period to review study conduct and compliance with the protocol, standard operation procedure, and the applicable regulatory requirements. The trial audit may be performed on a separate form.

### Ethics and dissemination

#### Ethics approval and consent to participate

The study conforms to the Declaration of Helsinki. Ethical approval has been respectively granted by (1) the Ethics Committee of the Zhongshan Ophthalmic Center, Sun Yat-sen University, China (ID: 2021KYPJ135); (2) the Ethics Committee of The First Affiliated Hospital of Guangxi Medical University (ID: 2021KYPJ112); and (3) the Ethics Committee of Shenzhen Eye Hospital (ID: 2021KYPJ001-01). The study has been registered at ClinicalTrials.gov (Trial registration number: NCT05136950) on November 1, 2021. All potential participants’ parents (or legal guardians) are required to sign an informed consent form before participating in the research, and the participants should re-consent when they reach 18.

#### Protocol amendments

Protocol amendments will be discussed and decided by the principal investigator and DMC. The ethical committee will be notified and its approval will be sought. Deviations from the protocol will be fully documented, using a report form.

#### Confidentiality

During the trial, personal information and data will be stored in the EDC system. Only authorized personnel in the research team can access the relevant data. Data used for statistical analysis are de-identified.

#### Dissemination plans

The interim analysis and final findings of the study will be published in peer-reviewed journals and disseminated by relevant national and international scientific conferences.

## Discussion

Pediatric cataract is the leading cause of avoidable childhood blindness worldwide. Treatment of pediatric cataract remains challenging because of the extremely high incidence of glaucoma-related AEs. Our prospective interventional case series have proved that compared to ciliary sulcus secondary IOL implantation, in-the-bag IOL implantation reduced AEs and yielded better IOL centration and VA for pediatric aphakia. Based on this research, we plan to conduct a multi-center RCT to further investigate the safety and efficacy of two approaches of IOL implantation in pediatric aphakia. The results will provide high-quality evidence for the clinical guidelines for the treatment of pediatric aphakia.

## Trial status

This is version 1.3 of the protocol, dated November 30, 2021. The first patient was randomized on December 14, 2021. Recruitment is predicted to continue until July 30, 2023. The last follow-up is expected to be completed on July 30, 2033. At the time of submission, participant recruitment is still ongoing.

## Data Availability

The dataset of this study and consent materials will be available from the corresponding author upon reasonable request.

## Abbreviations

AEs: Adverse events
IOL: Intraocular lens
VA: Visual acuity
IOP: Intraocular pressure
ETDRS: Early Treatment Diabetic Retinopathy Study
AS-OCT: Anterior segment optical coherence tomography
VCDR: Vertical cup-to-disc ratio
IQR: Interquantile range
ITT: Intention-to-treat
PP: Per-protocol
GEE: Generalized estimated equation
HR: Hazard ratio
DMC: Data monitoring committee
